# The association between current smoking and binge drinking among adults: A systematic review and meta-analysis of cross-sectional studies

**DOI:** 10.3389/fpsyt.2022.1084762

**Published:** 2023-01-18

**Authors:** Leila Molaeipour, Maryam Ghalandari, Hajar Nazari Kangavari, Zeinab Alizadeh, Elahe Jafari, Fatemeh Gholami, Neda Ghahremanzadeh, Shiva Safari, Vahideh Mohseni, Masoumeh Shahsavan, Seyed Abbas Motevalian

**Affiliations:** ^1^Department of Epidemiology, School of Public Health, Iran University of Medical Sciences, Tehran, Iran; ^2^Department of Epidemiology and Biostatistics, School of Public Health, Shahid Sadoughi University of Medical Sciences, Yazd, Iran; ^3^Social Determinants of Health Research Center, Research Institute for Prevention of Non-communicable Diseases, Qazvin University of Medical Sciences, Qazvin, Iran; ^4^Research Center for Addiction and Risky Behaviors (ReCARB), Psychosocial Health Research Institute (PHRI), Iran University of Medical Sciences, Tehran, Iran

**Keywords:** binge drinking, smoking, systematic review, meta-analysis, alcohol drinking

## Abstract

**Background:**

The substantial increasing trend of binge drinking is a global alarm. Our aim was to undertake a systematic review and meta-analysis of cross-sectional studies to explore the association of current smoking with binge drinking among adults.

**Methods:**

We systematically searched Web of Knowledge; PubMed; Scopus; Embase and Ovid (MEDLINE, EMBASE, PsycARTICLES, PsycINFO, PsycEXTRA, and PsycTests) (from inception to 27 May 2020) databases to identify cross-sectional studies of the association between current smoking and binge drinking. Study screening, data extraction, and methodological quality assessment were all carried out by two independent authors. Adjusted odds ratio (AOR) was pooled with 95% confidence intervals (CI) using random effects model in the meta-analysis, followed by the investigation of the heterogeneity *via Q*-test and *I*^2^ statistic. We assessed publication bias using a funnel plot, the Egger’s, and Begg’s tests.

**Results:**

We identified 3,171 studies and included nine cross-sectional studies with 64,516 participants. A significant association was found between current smoking and binge drinking among both genders (AOR = 2.97; 95% CI = 1.98 to 4.45; *I*^2^ = 90.5%). Subgroup analysis showed that this association among women, men, Caucasians, and Asians/Africans were (AOR = 3.68; 95% CI = 1.03 to 13.18; *I*^2^ = 98.9%), (AOR = 2.53; 95% CI = 1.87 to 3.42; *I*^2^ = 73.1%), (AOR = 1.36; 95% CI: 1.01–1.83, *I*^2^ = 47.4%), and (AOR = 3.93; 95% CI: 2.99–5.17, *I*^2^ = 61.3%), respectively. There was no evidence of publication bias.

**Conclusion:**

Current smoking is associated with binge drinking and can be used for identifying and screening binge drinkers. Moreover, this association is stronger among men, and Asians/Africans. This meta-analysis estimation was limited to English-language studies, and the full text of about 3.5% of reports for retrieval was not found, then generalization of the results should be done with caution.

## 1. Introduction

Binge alcohol drinking in adults is a preventable major public health problem (1, 2). Binge drinking is one of the most common, high-cost, and deadly patterns of heavy episodic alcohol use (1). It is defined as “a pattern of drinking that brings a person’s blood alcohol concentration to 0.08 g/dl or above. This typically happens when men consume 5 or more drinks or women consume 4 or more drinks in about 2 h” (1). Binge drinking as a dangerous act mainly leads to brain damage, intentional and unintentional injuries (such as car crashes, falls, burns, alcohol poisoning, suicide, homicide, intimate spouse violence, sexual assault, etc.), chronic diseases (such as liver or colon cancers, high blood pressure, stroke and other heart diseases), and worsening of comorbidities (1, 3). The substantial increasing trend in binge drinking among both the middle-aged and the elderly is a global alarm (4, 5). A meta-analysis showed that on average the prevalence of binge drinking is increasing by 0.72% per year (4).

Therefore, many scientists have recommended that binge drinking behaviors be screened in adults to minimize harm (6). Understanding the determinants of binge drinking is essential to implementing an alcohol harm reduction policy. Hence, various observational studies have been conducted to assess the effect of several protective or risk factors such as the existence of various physical or mental diseases, demographic factors, annual income, alcohol prices policy, religious or ethnicity diversity, and different drug use patterns (2, 5, 7, 8).

Smoking as an important variable in relation to binge drinking, is examined in different studies. Some cross-sectional studies have shown that in the adult population, current smokers are more likely to report binge drinking than non-smokers. However, the effect size of current smoking on binge drinking is contradictory in previous studies. While some studies have estimated the strength of the association between binge drinking and current smokers to be modest, other studies have estimated a 5–14 times higher chance of binge drinking among current smokers compared to non-smokers in the adult population (9–13).

Although cross-sectional studies may not establish a causal or temporal relationship between current smoking and binge drinking, they are nevertheless important. Despite their shortcomings, cross-sectional studies can prove whether there is an association between current smoking and binge drinking and whether these associations are substantive enough to consider current smoking for screening purposes.

Because of the inconsistency in the results of studies related to association of current smoking and binge drinking, we carried out a systematic review and meta-analysis of cross-sectional studies to explore this association among adult population.

## 2. Materials and methods

We followed the Meta-analyses Of Observational Studies in Epidemiology (MOOSE) Checklist for reporting this review. A copy of filled MOOSE checklist is attached in supporting information section (Supplementary material 1).

### 2.1. Search strategies and eligibility criteria

Cross-sectional studies investigating the association between current smoking and binge drinking among adults (18 years and over) were included despite gender, nationality, race, religion, or publication date. Papers published in non-English languages were excluded. Studies that were conducted in specific populations such as pregnant or breastfeeding women, or people with various diseases such as AIDS, metabolic disorders, etc., were also excluded. In addition, studies in which the effect of age was not adjusted were excluded from the study.

Binge drinking is defined as “a pattern of drinking that brings a person’s blood alcohol concentration to 0.08 g/dl or above. This typically happens when men consume 5 or more drinks or women consume 4 or more drinks in about 2 h” (2). Current smoking is defined as use of any type of smoked tobacco product on a daily or occasional basis (14). All studies that assessed the association between the past month and the past year binge drinking with current smoking were included in this study.

This study is part of a larger systematic review on the relationship between all types of smoking and alcohol consumption. The search strategy was a combination of the selected keyword sets within the titles, abstracts and keywords. All major electronic databases including Web of Knowledge; PubMed; Scopus; Embase and Ovid (Ovid MEDLINE, Ovid EMBASE, Ovid PsysARTICLES, Ovid PsyscINFO, Ovid-PsycEXTRA, and Ovid PsysTests) were searched from database inception to 27 May 2020. Boolean operators and truncations were different according to the databases. Relevant MeSH and -Emtree terms were included when searching the PubMed and Embase databases, respectively. Details of all searches can be found in the supporting information section (Supplementary material 2).

To manage the search results of all databases, protocols for study screening, data extraction and quality assessment were first developed by the research team. All protocols were clear and concise and were refined during the pilot study. A training workshops was held for the research team members and screening of 20 studies was done on a trial basis. During study, meetings with members of the research team were repeated as necessary. All search results of databases were combined using Excel version 2016, and duplicates were deleted. Each of the retrieved studies were screened by two independent reviewers based on the title and abstract and were classified into three groups: related, unrelated and undetectable. Undetectable studies were re-reviewed by a senior reviewer. The full text of all selected studies was searched from databases, and when necessary, from other sources such as searching ResearchGate and emailing corresponding authors. The screened positive studies were assessed based on their full text to figure out eligible cross-sectional studies on the association current smoking and binge drinking. Five reviewers [LM (50%), ZA (50%), MG (40%), HK (40%), and EJ (20%)] were involved in finding eligible studies. Any discrepancies between reviewers at each step were resolved by discussion until consensus was reached, otherwise a senior reviewer judged the case.

### 2.2. Data extraction and quality assessment

Data extraction was carried out by 8 authors [LM (20%), ZA (30%), MG (30%), HK (20%), EJ (25%), NG (25%), FG (25%), and SS (25%)] using a previously piloted excel sheet. The following key data were extracted: first author’s name, publication year, country, participant characteristics (age, gender, and nationality), sample size, type, and method of smoking and drinking measurement, smoking and alcohol definitions, and adjusted odds ratio (AOR) with 95% confidence interval (CI).

The methodological quality of the included studies was explored by two independent authors. Five authors [LM (10%), SS (20%), NG (25%), MS (20%), and VM (25%)] did the quality assessment using the Newcastle-Ottawa Scale adapted for cross-sectional studies (15). This scale assigns up to ten stars for selection (maximum 5 stars), comparability (maximum 2 stars), and outcome (maximum 3 stars) to the cross-sectional studies. The studies with seven or more stars were considered to have a low-risk bias, and those with six or less stars were considered as high risk. Any disagreement between reviewers at each step was resolved by discussion.

### 2.3. Statistical analysis

Adjusted measures of effect were extracted from each study. Effect measures were extracted as adjusted odds ratios (AOR), with 95% confidence intervals (CI). Random-effects meta-analysis and the inverse variance weighting method were adopted to estimate the pooled measures of effect across studies. Heterogeneity was investigated using *Q*-test (16) and *I*^2^ statistic (17). Sensitivity analysis was employed to investigate the heterogeneity sources between studies. Also, a random-effects meta-regression was runed in the studies reporting the association in both genders to explore whether effect size varied by study sample size, data collection year (2007–2013, 2014–2020), and nationality (American and European, Asians and Africans). The Egger’s (18), and Begg’s (19) tests, and a funnel plot were applied to assess publication bias. Stata software (version 14.1, StataCorp, College Station, TX, USA) was used to perform analyses.

## 3. Results

### 3.1. Overview of the included studies

The systematic search of electronic databases retrieved 190,040 references until 27 May 2020, and after removing duplicate references, 56,718 studies remained. After reviewing the titles and abstracts, 53,547 studies that were not related to the association of smoking and alcohol drinking were excluded. For the 3,171 remained studies, full texts were reviewed and 2,692 studies were excluded for the following reasons: 2,243 studies were not related to the association between smoking and drinking, 205 studies were not in English, 123 studies did not report the effect size, and 120 studies’ full texts were not found. In general, 480 studies related to smoking and alcohol drinking were identified. Of these, 365 studies were excluded for the following reasons: in 220 studies smoking was outcome, in 127 studies, study population aged less than 18 years, and 18 studies were focused on patients (HIV/AIDS, ADHD, epilepsy, etc.) or individuals with specific conditions like pregnancy or breastfeeding.

**FIGURE 1:**
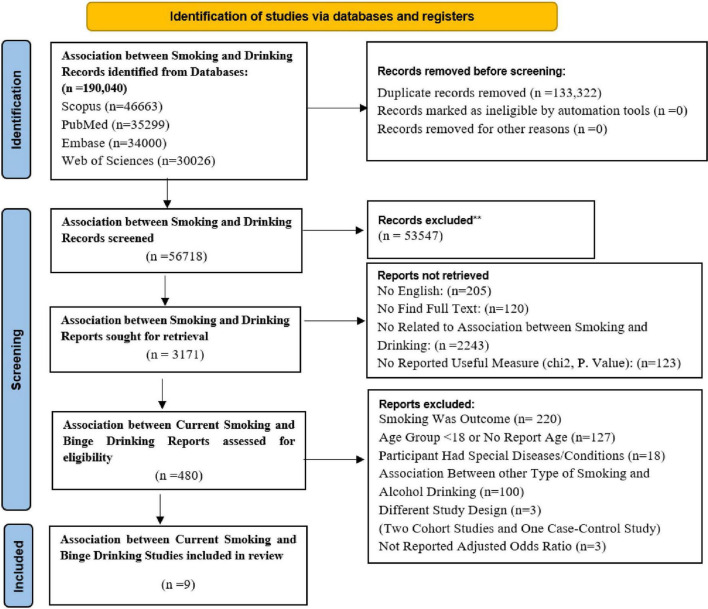
Study selection.

Among the remaining 115 studies, studies related to current smoking and binge drinking were selected, and 106 studies (complete list is available at supporting information section Supplementary material 3) were excluded for the following reasons: in 100 studies the relationship between other types of smoking and alcohol was investigated, in 3 studies the study design was not cross-sectional (two cohorts and one case-control study), and in 3 study the adjusted odds ratios were not reported. Eventually, 9 studies were eligible for inclusion in the current systematic review (Figure 1).

Nine cross-sectional studies reported data on the association between current smoking and binge drinking among adults, published between 2007 and 2018. Five studies were done by researchers from the United States (5, 9, 12, 13, 20), one study was from the United States and northern Europe (21), the other three studies were from Hong Kong (10), South Korea (22), and South Africa (11). The summary of included studies, the odds ratios and 95% confidence intervals for crude and adjusted estimates, and description confounders are presented in Tables 1, 2.

**TABLE 1:** Summary of studies results.

References	Country	Age group	Gender	Sample	Binge drinking	Estimate	NOS*		
							Sel	Com	Out
Owolabi et al. (11)	South Africa	18–75	Both	998	1 month	Adjusted	***	**	**
Tsai et al. (12)	USA	18–44	Female	21953	1 month	Adjusted	***	**	**
Becerra et al. (13)	USA (Chinese)	> = 18	Both	3576	12 month	Adjusted	***	**	**
	USA (Filipino)			1638					
	USA (South Asian)			1352					
	USA (Japanese)			1175					
	USA (Korean)			2298					
	USA (Vietnamese)			2800					
Bartoli et al. (21)	USA and Northern Europe	>18	Both	654	Unknown	Adjusted	****	**	**
Parikh et al. (5)	USA	> = 65	Both	4815	1 month	Adjusted	****	**	**
			Male	2270		Adjusted			
			Female	2545		Crude			
Kim et al. (10)	Hong Kong (Chinese)	18–70	Both	9896	1 month	Adjusted	****	**	**
			Male	4950					
			Female	4946					
Blazer and Wu (9)	USA	> = 50	Male	4952	1 month	Adjusted	***	**	**
			Female	6001					
Gubner et al. (20)	USA	18–25	Both	563	1 Month	Adjusted	*****	**	**
Kim and Sang (22)	Korea	>20	Both	1845	Unknown	Adjusted	****	**	**

Sel, selection; Com, comparability; Out, outcome. Adjusted means controlled for one or more of the following factors: age, gender, race, educational level, income, ethnicity, mental disorder, drug abuse, marital status, body mass index, employment status. NOS, the Newcastle-Ottawa Quality statement manual. Each * means one score in the Newcastle-Ottawa scale.

**TABLE 2:** Description of current smoking and binge-drinking definition, report unadjusted and adjusted odds ratios with 95% confidence intervals and description confounders.

References	Exposure definition	Measuring tool	Outcome definition	Measuring tool	Unadjusted OR	Adjusted OR	Covariate
Owolabi et al. (11)	NE	NE		WHO STEP wise Questionnaire	7.15 (4.72–10.78)	6.50 (3.50–11.90)	Age, sex, income
Tsai et al. (12)	NE	NE	Have had at least five drinks on any one occasion during the previous 30 days.	The BRFSS Method	NR	1.30 (1.25–1.36)	Age
Becerra et al. (13)	NE	NE	Binge-drinking is defined as 5 or more drinks for men and 4 or more drinks for women per occasion binge-drinking in the past 30 days.	SAQ			Age, sex, marital status.
Chinese-American					3.01 (2.31–3.93)	2.86 (1.89–4.33)	
Filipino-American					2.72 (2.17–3.42)	3.21 (2.05–5.05)	
South Asian-American					2.11 (1.37–3.18)	1.89 (0.95–3.75)	
Japanese-American					3.47 (2.70–4.46)	4.26 (2.38–7.64)	
Korean-American					2.03 (1.41–2.90)	2.70 (1.44–5.07)	
Vietnamese-American					2.21 (1.69–2.88)	8.43 (3.73–19.06)	
Bartoli et al. (21)	Habits of smoking nicotine cigarettes during the last 30 days.	SAQ	Binge-drinking is defined as 5 or more drinks for men and 4 or more drinks for women per occasion binge-drinking in the past 30 days.	SAQ	1.90 (1.36–2.67)	1.04 (0.74–1.46)	Age, sex, specific place of recruitment.
Parikh et al. (5)							
Both	NE	SAQ	Binge-drinking is defined as 5 or more drinks for men and 4 or more drinks for women per occasion binge-drinking in the past 30 days.	SAQ	2.32 (1.77–3.00)	1.59 (1.18–2.15)	Age, sex, ethnic, BMI, occupation, income.
Male					2.32 (1.61–3.29)	1.63 (1.10–2.42)	
Female					2.56 (1.68–3.83)	NR	
Kim et al. (10)	NE	SAQ	NE	DSM-IV criteria			Age, education, marital status, employment
Both					14.73 (11.34–19.22)	6.03 (1.5–24.04)	
Male					3.60 (3.00–4.20)	3.00 (2.30–3.80)	
Female					14.80 (10.5–20.9)	12.30 (8.60–17.70)	
Blazer and Wu (9)	NE	SAQ	Binge-drinking is defined as 5 or more drinks for men and 4 or more drinks for women per occasion binge-drinking in the past 30 days.	SAQ			Age, race/ethnicity, educational level, marital status, employed, income, serious psychological distress. Use of illicit drugs, non-medical use of prescription drugs, Survey year.
Male					NR	2.90 (2.41–3.61)	
Female					NR	3.20 (2.41–4.33)	
Gubner et al. (20)	Total cigarettes smoked each day in the past month	Timeline follow back (TLFB)	Binge-drinking is defined as 5 or more drinks for men and 4 or more drinks for women per occasion binge-drinking in the past 30 days.	Timeline follow back (TLFB)	NR	1.60 (1.00–2.70)	Age, sex, ethnicity, years of education, household income.
Kim and Sang (22)	NE	SAQ	Binge-drinking is defined as 5 or more drinks for men and 4 or more drinks for women per occasion binge-drinking.	KNHANES IV	5.35 (4.64–6.16)	4.95 (4.25–5.77)	Age, sex, marital status. income, education and occupation.

*SAQ, Self-Administered Questionnaire; NE, no explained; NR, no report.

Table 1 summarizes the study characteristics of the included studies. The total number of participants in the studies was 64,516 (each study sample size ranged from 563 to 21,953). In one of the studies, the association was reported for six Asian-American subpopulations, separately (Chinese, Filipino, South Asian, Japanese, Korean, and Vietnamese) (13). Five studies reported the association in all subjects without separating by gender (11, 13, 20–22), two studies reported association separately for male, female, and total subjects (5, 10), one study reported association separately for male and female subjects without reporting for total sample (9), finally one study was female only (12).

### 3.2. The result of meta-analysis

**FIGURE 2:**
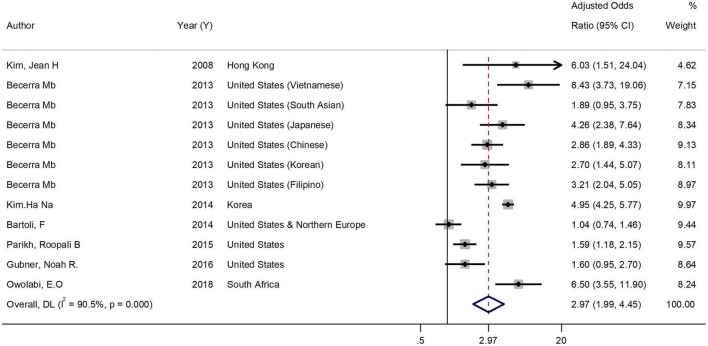
Forest plot of the association between binge drinking and current smoking. Weights are from random-effects model.

**FIGURE 3:**
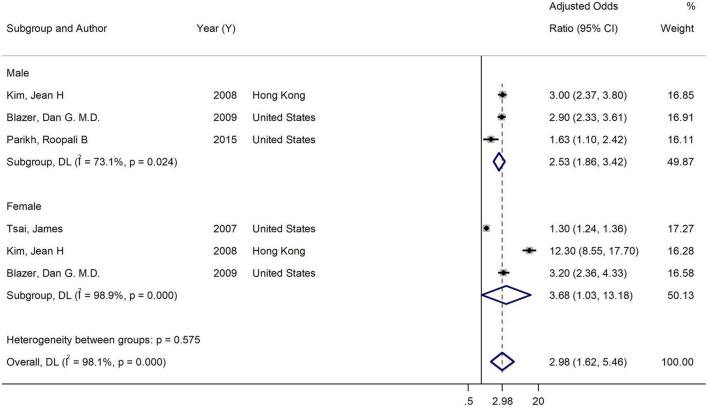
Forest plot of the association between binge-drinking and current smoking by gender. Weights and between-subgroup heterogeneity test are from random-effects model.

Overall AOR for both genders, based on pooling the results of 7 studies, was 2.97 (95% CI = 1.98 to 4.45). Heterogeneity was assessed using the *Q*-test and the *I*^2^ statistic. Figure 2 shows a significant heterogeneity (*Q*-test = 115.88, *P* < 0.001; *I*^2^ = 90.5%) among studies addressing association between current smoking and binge drinking. The result of the subgroup analysis of gender found that gender explains to some extent the high level of heterogeneity [Female: 3.68 (95% CI = 1.03 to 13.18; *I*^2^ = 98.9%) vs. (Male: 2.53 (95% CI = 1.87 to 3.42; *I*^2^ = 73.1%)] (Figure 3).

**FIGURE 4:**
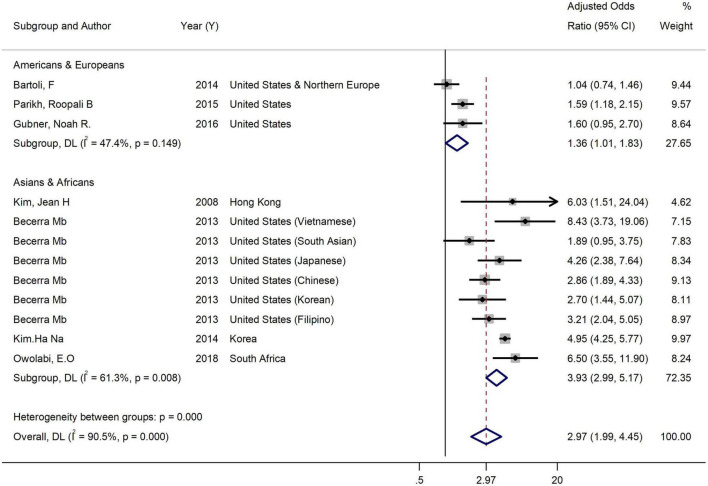
Forest plot of the association between binge drinking and current smoking by nationality. Weights and between-subgroup heterogeneity test are from random-effects model.

Sub-group analysis in the studies reporting the association in both genders by nationality showed that the adjusted effect size among American and European population was 1.36 (95% CI: 1.01–1.83, *I*^2^ = 47.4%), while it was 3.93 (95% CI: 2.99–5.17, *I*^2^ = 61.3%) among Asians and Africans. So, nationality is another characteristic that could explain heterogeneity (Figure 4).

Table 3 shows the random effect meta-regression results. The nationality influenced the magnitude of the effect size between current smoking and binge drinking. Effect size was larger among samples from Asians and Africans versus American and European. Nationality explained alone about 78% of between-studies variance.

**TABLE 3:** Result of random effect meta-regression on the association between current smoking and binge drinking in both sexes.

Variables	Univariable associations (95% CI)	Multivariate associations (95% CI)	Bivariable adjusted *R*^2^ (%)
**Year**			
2007–2013	1.00	1.00	−0.91
2014–2020	0.68 (0.29–1.57)	2.14 (0.77–5.96)	
**Nationality**			
Americans and Europeans	1.00	1.00	78.63
Asians and Africans	2.88 (1.68–4.93)*	5.19 (1.85–14.56)*	
Sample size	1.00 (0.99–1.00)	1.00 (0.99–1.00)	−7.15

*Significant at 0.05 level.

**FIGURE 5:**
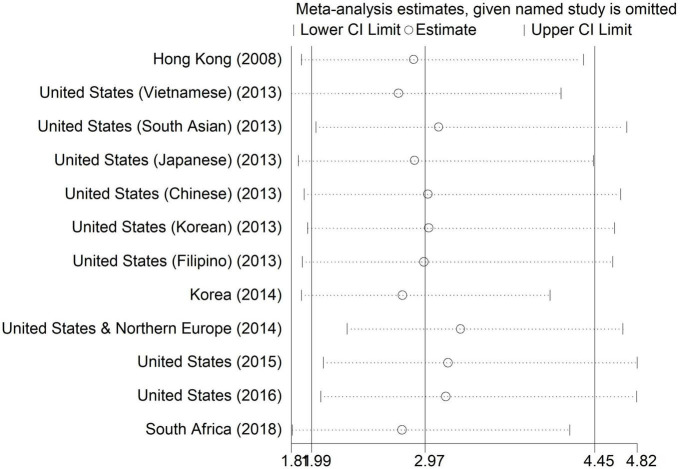
Metaninf plot of the association between current smoking and binge drinking in both sexes.

Additionally, to examine heterogeneity between studies, the Metaninf commands were used to show the effect of removing each study on the overall result, in both sexes. According to the results, the exclusion of none of the studies had a significant role in changing the AOR. Among them, the exclusion of the study by Bartoli et al. (21) had the largest effect on the estimated AOR (3.28) (Figure 5 and Supplementary material 4).

**FIGURE 6:**
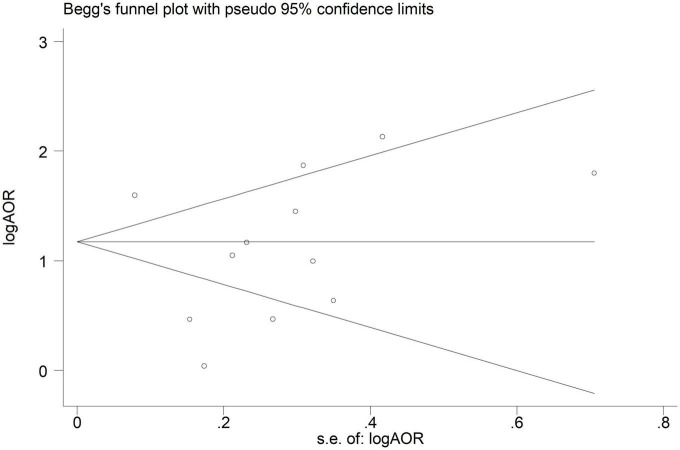
Begg’s funnel plot of included studies assessing the publication bias in studies addressing the association between binge-drinking and current smoking.

The risk of bias assessment of the studies listed in Table 1 are based on the Newcastle-Ottawa scale (15). All 9 studies had a low risk of bias. The possibility of publication bias was explored using a funnel plot, Egger and, Begg statistical tests. Based on Begg’s funnel plot results (Figure 6), the studies are symmetrically scattered on both sides of the horizontal line, and there is no evidence of publication bias in the studies. Also, the results of the Egger and Begg statistical test confirmed this finding. The Egger’s test (*P* = 0.273) and Begg’s test (*P* = 0.457) were statistically non-significant.

## 4. Discussion

In this systematic review and meta-analysis of cross-sectional studies, the association between current smoking and binge drinking among adults was significant with a pooled AOR of 2.97 (95% CI = 1.98 to 4.45). To the best of our knowledge, our study is one of the first attempts to provide a summary estimate of the effects size of the association between current smoking and binge drinking.

The strong association between current smoking and binge drinking suggests that these behaviors may have common cause or that the use of a substance affects the use or initiation of another substance. This is particularly important in the old adults, as binge drinking in elderly is more easily missed in actual clinical practice than in other groups (9). Although the effects of binge drinking on morbidity and mortality in the elderly are undeniable, they remain largely unrecognized, and patients are denied assistance (1, 3, 5). Evidence suggests that healthcare visits in these individuals are less likely to diagnose binge drinking because they are more focused on identifying and managing chronic medical conditions (23). Moreover, it is likely that healthcare workers be biased and eliminated several aspects of alcohol screening in old adults (9). In this context, current smoking, possibly with less bias, can play an important role in screening elderly binge drinkers.

In limited previous studies, relationships between some sociodemographic factors such as age, gender, education and income with binge drinking have been observed (24–26). Although the studies that were adjusted for at least one of these factors were included in our meta-analysis, the sensitivity analysis indicated that heterogeneity between studies may be due to the role of gender and nationality.

Our results indicated a strong association between current smoking and binge alcohol in both genders. Female current smokers were approximately 4 times more, and male current smokers were about 3 times more odds of binge drink than non-smokers. Binge drinking is nearly twice as common among men than women (1). The results of the heterogeneity test, *I*^2^ statistic, 73.1%, showed that the pooled AOR observed between current smoking and binge drinking was more stable in men. But the large heterogeneity in AOR reported in women, due to the study conducted in Hong Kong with AOR:12.30, may indicate the role of ethnicity or genetic in this stark difference. This is also evident in subgroup analysis based on nationality.

As presented in the results, we found a strong positive association between current smoking and binge drinking among Asian or Africans. The effect size of the association in Asian or African nationalities was 3.93, while it was 1.36 among the Caucasian population. The Asian subgroup included people from Hong Kong, Korea, and Asian immigrants to the United States. The homogeneity of AORs reported in these populations may be related to the ethnic similarity of these individuals.

There are many reports of the effect of genes related to subjective response on alcohol drinking (27). Researchers are trying to elucidate the mechanism by which genes ultimately cause differences in alcohol drinking through behavioral variables (28). They speculate that the acute or chronic effects of smoking on subjective responses to alcohol can play a role in this relationship (29). A study of Australian twins confirms a positive genetic correlation between regular smoking and the risk of alcohol dependence, which is significant even after adjusting for demographic and personality variables, and a history of another psychopathology (29). Also, the genetic polymorphism differences indicate variation in genetic susceptibility to alcohol drinking in the diverse ethnic populations (27, 30). In a study conducted in various parts of China, it was shown that Tibetan regions have the highest frequency of risk alleles for heavy drinking (27). Therefore, it seems that genetic predisposition toward unhealthy lifestyles may explain a part of the difference observed between different nationalities observed in the present study.

There are limitations in this study that may be the source of biases and should be considered in the interpretation of the results and addressed in future systematic reviews. First, this systematic review and meta-analysis study focused on English-language studies and 205 studies published in other languages were excluded. Second, despite extensive efforts by the research team to access the full text of studies, emailing authors and searching ResearchGate, 120 studies remained without full text. It is important to note that the broad search strategy was to examine the overall association of smoking and drinking, not the current smoking and binge drinking, which is fully described in the Section “2. Materials and methods.” Third, gray literature, which are important sources to minimize the risk of omitting related sources, was not included in this study. Notwithstanding these shortcomings, in this study, an extensive search strategy was used on the important bibliography databases (Web of science, PubMed, Scopus, Embase) and medical research platform Ovid (Ovid MEDLINE, Ovid EMBASE, Ovid PsysARTICLES, Ovid PsyscINFO, Ovid-PsycEXTRA, and Ovid PsysTests) to find all relevant published studies.

## 5. Conclusion

This systematic review and meta-analysis measured the association between current smoking and binge drinking. We found that current smokers are almost three folds more likely to report binge drinking than who never smoke. Subgroup analysis showed that the association was more than three and a half times greater among female current smokers and nearly fourfold among Asian/African current smokers. Therefore, it seems that current smoking can play an important role in identifying and screening binge drinkers.

## Data availability statement

The original contributions presented in this study are included in the article/Supplementary material, further inquiries can be directed to the corresponding author.

## Author contributions

SM and LM presented the idea of this study, developed the search strategies, and performed a meta-analysis. LM, ZA, MG, HK, EJ, NG, FG, SS, MS, and VM performed data screening, data extraction, and critical appraisal of studies. LM drafted the manuscript. All authors discussed the results and contributed to the final manuscript.
