# Supplementary material for: The association between current smoking and binge drinking among adults: A systematic review and meta-analysis of cross-sectional studies
Source: Front Psychiatry. 2023 Jan 18;13:1084762. doi: 10.3389/fpsyt.2022.1084762 (PMC9889925; doi:10.3389/fpsyt.2022.1084762)
Supplement: Supplementary file 2 [file Table_2.docx]

**Search Strategy**

The following databases were searched until May 27, 2020. The search strategy of each database and search results are as follows:

| **Databases** | **Search Strategy** | **Result** |
| --- | --- | --- |
| **Scopus** | (TITLE-ABS-KEY(smok*) OR TITLE-ABS-KEY(Tobacco*) OR TITLE-ABS-KEY(Nicotine) OR TITLE-ABS-KEY(cigar*)) AND (TITLE-ABS-KEY(drink*) OR TITLE-ABS-KEY("alcohol *")) AND (TITLE-ABS-KEY("Epidemiologic Stud*") OR TITLE-ABS-KEY("epidemiological stud*") OR TITLE-ABS-KEY(cohort*) OR TITLE-ABS-KEY( "longitudinal stud*") OR TITLE-ABS-KEY("prospective stud*") OR TITLE-ABS-KEY("retrospective stud*") OR TITLE-ABS-KEY( "Case-Control Stud*") OR TITLE-ABS-KEY("case-cohort stud*") OR TITLE-ABS-KEY("cross-sectional stud*") OR TITLE-ABS-KEY(survey)) | **46663** |
| **PubMed** | ((drinking behavior [MeSH Terms] OR Alcoholism [MeSH Terms] OR Alcoholics [MeSH Terms] OR drink*[Title/Abstract] OR alcohol*[Title/Abstract]) AND (smoking[MeSH Terms] OR smokers[MeSH Terms] OR Tobacco Use[MeSH Terms] OR Nicotine[MeSH Terms] OR smok*[Title/Abstract] OR cigar*[Title/Abstract] OR tobacco[Title/Abstract] OR Nicotine[Title/Abstract]) AND (Epidemiologic Studies[MeSH] OR epidemiological stud*[Title/Abstract] OR epidemiologic stud*[Title/Abstract] OR cohort*[Title/Abstract] OR longitudinal stud*[Title/Abstract] OR prospective stud*[Title/Abstract] OR retrospective stud*[Title/Abstract] OR Case Control Stud*[Title/Abstract] OR case cohort stud*[Title/Abstract] OR cross sectional stud*[Title/Abstract] OR survey[Title/Abstract] OR Observational Study[pt])) | **35299** |
| **Ovid** | ((Drinking Behavior* or Alcoholism or drink* or alcohol*) and (smok* or tobacco* or cigar* or nicotine or Smoking or "Tobacco Use*") and ("epidemiological stud*" or "longitudinal stud*" or "prospective stud*" or "retrospective stud*" or "Case Control Stud*" or "case cohort stud*" or "cross sectional stud*" or survey or cohort*)).ti,ab,hw | **44052** |
| **Embase** | )('alcohol'/syn OR alcohol*:ti,ab OR 'alcohol consumption'/syn OR 'drinking behavior'/syn OR 'drink*':ti,ab OR 'alcoholism'/syn OR 'alcohol abuse'/syn) AND ('tobacco use'/syn OR 'tobacco*':ti,ab OR 'smoking'/syn OR 'smok*':ti,ab OR 'nicotine'/syn OR 'nicotine':ti,ab OR 'cigar*':ti,ab) AND ('epidemiological stud*':ti,ab OR 'epidemiologic stud*':ti,ab OR 'cohort analysis'/syn OR 'cohort*':ti,ab OR 'longitudinal stud*':ti,ab OR 'prospective stud*':ti,ab OR 'retrospective stud*':ti,ab OR 'case control study'/syn OR 'case control stud*':ti,ab OR 'case-cohort stud*':ti,ab OR 'cross-sectional stud*':ti,ab OR 'survey':ti,ab OR 'observational study'/syn OR 'observational stud*':ti,ab ) AND [1-1-0001]/sd NOT [27-5-2020]/sd( | **34000** |
| **Web of Science** | TS=(drink* OR alcohol*) AND TS=(smok* OR cigar* OR tobacco OR nicotine) AND TS=((epidem* OR longitudinal OR prospective OR retrospective OR "case control" OR "case cohort" OR "cross sectional" OR survey OR cohort) AND stud*))  Indexes=SCI-EXPANDED, SSCI, A&HCI, CPCI-S, CPCI-SSH, BKCI-S, BKCI-SSH, ESCI, CCR-EXPANDED, IC Timespan=All years) | **30026** |
