# Supplementary material for: The association between current smoking and binge drinking among adults: A systematic review and meta-analysis of cross-sectional studies
Source: Front Psychiatry. 2023 Jan 18;13:1084762. doi: 10.3389/fpsyt.2022.1084762 (PMC9889925; doi:10.3389/fpsyt.2022.1084762)
Supplement: Supplementary file 3 [file Table_3.docx]

| **Excluded Studies in Qualitative Synthesis**  **Association Between Current Smoking and Binge Drinking** |
| --- |
| **The type of alcohol drinking was different**   1. Bobak M, McKee M, Rose R, Marmot M. Alcohol consumption in a national sample of the Russian population. Addiction. 1999 Jun;94(6):857-66. 2. Potenza MN, Steinberg MA, Wu R. Characteristics of gambling helpline callers with self-reported gambling and alcohol use problems. Journal of Gambling Studies. 2005 Sep;21(3):233-54. 3. Friedman GD, Tekawa I, Klatsky AL, Sidney S, Armstrong MA. Alcohol drinking and cigarette smoking: an exploration of the association in middle-aged men and women. Drug and alcohol dependence. 1991 May 1;27(3):283-90. 4. Gubner NR, Delucchi KL, Ramo DE. Associations between binge drinking frequency and tobacco use among young adults. Addictive behaviors. 2016 Sep 1; 60:191-6. 5. Jackson KM, Sher KJ, Wood PK, Bucholz KK. Alcohol and tobacco use disorders in a general population: short-term and long-term associations from the St. Louis Epidemiological Catchment Area Study. Drug and alcohol dependence. 2003 Sep 10;71(3):239-53. 6. Blay SL, Fillenbaum GG, Andreoli SB, Gastal FL. Correlates of lifetime alcohol misuse among older community residents in Brazil. International psychogeriatrics. 2009 Apr;21(2):384-91. 7. Moore RS, Cunradi CB, Duke MR, Ames GM. Dimensions of problem drinking among young adult restaurant workers. The American journal of drug and alcohol abuse. 2009 Jan 1;35(5):329-33. 8. Pirkola SP, Poikolainen K, Lönnqvist JK. Currently active and remitted alcohol dependence in a nationwide adult general population—results from the Finnish Health 2000 study. Alcohol and alcoholism. 2006 May 1;41(3):315-20. 9. Veerbeek MA, Ten Have M, van Dorsselaer SA, Voshaar RC, Rhebergen D, Willemse BM. Differences in alcohol use between younger and older people: results from a general population study. Drug and alcohol dependence. 2019 Sep 1; 202:18-23. 10. Rathod SD, Nadkarni A, Bhana A, Shidhaye R. Epidemiological features of alcohol use in rural India: a population-based cross-sectional study. BMJ open. 2015 Dec 1;5(12): e009802. 11. Midorikawa H, Tachikawa H, Aiba M, Arai T, Watanabe T, Tamiya N. Factors associated with high‐risk drinking in older adults: Evidence from a national survey in Japan. Geriatrics & Gerontology International. 2019 Dec;19(12):1260-7. 12. Santangelo OE, Provenzano S, Piazza D, Firenze A. SHORT PAPER Factors associated with risky consumption of alcohol in a sample of university students. Ann Ig. 2018; 30:502-8. 13. Ohida N, Otsuka Y, Kaneita Y, Nakagome S, Jike M, Itani O, Ohida T. Factors related to alcohol consumption among Japanese physicians. Asia Pacific Journal of Public Health. 2018 Apr;30(3):296-306. 14. Hirata ES, Nakano EY, Junior JA, Litvoc J, Bottino CM. Prevalence and correlates of alcoholism in community‐dwelling elderly living in São Paulo, Brazil. International Journal of Geriatric Psychiatry: A journal of the psychiatry of late life and allied sciences. 2009 Oct;24(10):1045-53. 15. Veerbeek MA, Ten Have M, van Dorsselaer SA, Voshaar RC, Rhebergen D, Willemse BM. Differences in alcohol use between younger and older people: results from a general population study. Drug and alcohol dependence. 2019 Sep 1; 202:18-23. 16. Lee YH, Chang YC, Liu CT, Shelley M. Correlates of alcohol consumption and alcohol dependence among older adults in contemporary China: results from the Chinese longitudinal healthy longevity survey. Journal of Ethnicity in Substance Abuse. 2020 Jan 2;19(1):70-85. 17. Williams JO, Bell NS, Amoroso PJ. Drinking and other risk taking behaviors of enlisted male soldiers in the US Army. Work. 2002 Jan 1;18(2):141-50. 18. Ryu SY, Crespi CM, Maxwell AE. Drinking patterns among Korean adults: results of the 2009 Korean community health survey. Journal of preventive medicine and public health. 2013 Jul;46(4):183. 19. Kuteesa MO, Weiss HA, Cook S, Seeley J, Ssentongo JN, Kizindo R, Ngonzi P, Sewankambo M, Webb EL. Epidemiology of alcohol misuse and illicit drug use among young people aged 15–24 years in fishing communities in Uganda. International journal of environmental research and public health. 2020 Apr;17(7):2401. 20. Rafferty E, Bonner WI, Code J, McBride K, Andkhoie M, Tikoo R, McClean S, Dell C, Szafron M, Farag M. Factors influencing risky single occasion drinking in Canada and policy implications. Archives of public health. 2017 Dec;75(1):1-1. 21. Katulanda P, Ranasinghe C, Rathnapala A, Karunaratne N, Sheriff R, Matthews D. Prevalence, patterns and correlates of alcohol consumption and its’ association with tobacco smoking among Sri Lankan adults: A cross-sectional study. BMC public health. 2014 Dec;14(1):1-0. 22. Gottlieb Hansen AB, Hvidtfeldt UA, Grønbæk M, Becker U, Søgaard Nielsen A, Schurmann Tolstrup J. The number of persons with alcohol problems in the Danish population. Scandinavian journal of public health. 2011 Mar;39(2):128-36. 23. Asciutto R, Lugo A, Pacifici R, Colombo P, Rota M, La Vecchia C, Gallus S. The particular story of italians' relation with alcohol: trends in individuals' consumption by age and beverage type. Alcohol and Alcoholism. 2016 May 1;51(3):347-53. 24. Burger M, Mensink GB, Bergmann E, Pietrzik K. Characteristics associated with alcohol consumption in Germany. Journal of studies on alcohol. 2003 Mar;64(2):262-9. 25. Weyerer S, Schäufele M, Eifflaender‐Gorfer S, Köhler L, Maier W, Haller F, Cvetanovska‐Pllashiniku G, Pentzek M, Fuchs A, van den Bussche H, Zimmermann T. At‐risk alcohol drinking in primary care patients aged 75 years and older. International journal of geriatric psychiatry. 2009 Dec;24(12):1376-85. 26. Mazas CA, Cofta-Woerpel L, Daza P, Fouladi RT, Vidrine JI, Cinciripini PM, Gritz ER, Wetter DW. At-risk drinking in employed men and women. Annals of Behavioral Medicine. 2006 Jun;31(3):279-87. 27. Gilbert PA, Marzell M. Characterizing a hidden group of at-risk drinkers: Epidemiological profiles of alcohol-use disorder diagnostic orphans. Substance use & misuse. 2018 Jul 3;53(8):1239-51. 28. Roberts W, Verplaetse T, Peltier MK, Moore KE, Gueorguieva R, McKee SA. Prospective association of e‐cigarette and cigarette use with alcohol use in two waves of the Population Assessment of Tobacco and Health. Addiction. 2020 Aug;115(8):1571-9. 29. Assanangkornchai S, Saunders JB, Conigrave KM. Patterns of drinking in Thai men. Alcohol and Alcoholism. 2000 May 1;35(3):263-9. 30. Bobo JK, Greek AA, Klepinger DH, Herting JR. Predicting 10-year alcohol use trajectories among men age 50 years and older. The American Journal of Geriatric Psychiatry. 2013 Feb 1;21(2):204-13. 31. Bellón JÁ, de Dios Luna J, King M, Nazareth I, Motrico E, GildeGómez-Barragán MJ, Torres-Gonzalez F, Montón-Franco C, Sánchez-Celaya M, Díaz-Barreiros MÁ, Vicens C. Predicting the onset of hazardous alcohol drinking in primary care: development and validation of a simple risk algorithm. British journal of general practice. 2017 Apr 1;67(657):e280-92. 32. Pengpid S, Peltzer K, Van der Heever H. Prevalence of alcohol use and associated factors in urban hospital outpatients in South Africa. International journal of environmental research and public health. 2011 Jul;8(7):2629-39. 33. Wartberg L, Kriston L, Thomasius R. Prevalence of problem drinking and associated factors in a representative German sample of adolescents and young adults. Journal of Public Health. 2019 Sep 30;41(3):543-9. 34. Kinner SA, Dietze PM, Gouillou M, Alati R. Prevalence and correlates of alcohol dependence in adult prisoners vary according to Indigenous status. Australian and New Zealand Journal of Public Health. 2012 Aug;36(4):329-34. 35. Mori H, Fukuda T. Prevalence patterns of alcohol consumption and factors associated with problematic drinking on remote islands of Okinawa, Japan: a cross-sectional study. Journal of Rural Medicine. 2020;15(2):50-6. 36. Jeon HJ, Hahm BJ, Lee HW, Hong JP, Bae JN, Park JI, Kim JK, Bae A, Park JH, Chung EK, Shin JH. Prevalence, correlates, and comorbidity of 12-Month tobacco dependence among ever-smokers in South Korea, during 1984-2001. Journal of Korean medical science. 2008 Apr 1;23(2):207-12. 37. Kendagor A, Gathecha G, Ntakuka MW, Nyakundi P, Gathere S, Kiptui D, Abubakar H, Ombiro O, Juma P, Ngaruiya C. Prevalence and determinants of heavy episodic drinking among adults in Kenya: analysis of the STEPwise survey, 2015. BMC Public Health. 2018 Nov;18(3):1-9. 38. Moore AA, Gould R, Reuben DB, Greendale GA, Carter MK, Zhou K, Karlamangla A. Longitudinal patterns and predictors of alcohol consumption in the United States. American journal of public health. 2005 Mar;95(3):458-64. 39. Morgen CS, Bové KB, Larsen KS, Kjær SK, Grønbæk M. Association between smoking and the risk of heavy drinking among young women: a prospective study. Alcohol & Alcoholism. 2008 May 1;43(3):371-5. 40. Ordóñez AE, Ranney R, Schwartz M, Mathews CA, Satre DD. Hazardous drinking among young adults seeking outpatient mental health services. Addiction science & clinical practice. 2016 Dec;11(1):1-7. 41. Philalai T, Rattanapan C, Laosee O. Alcohol consumption among older adults in northern Thailand. Journal of Health Research. 2017;31(2):99-107. 42. Jaeger GP, Mola CL, Silveira MF. Alcohol-related disorders and associated factors in a rural area in Brazil. Revista de Saúde Pública. 2018 Sep 17;52. 43. Fleming MF, Manwell LB, Barry KL, Johnson K. At-risk drinking in an HMO primary care sample: prevalence and health policy implications. American Journal of Public Health. 1998 Jan;88(1):90-3. 44. Maloney E, Hutchinson D, Burns L, Mattick R. Prevalence and patterns of problematic alcohol use among Australian parents. Australian and New Zealand journal of public health. 2010 Oct;34(5):495-501. |
| **The type of alcohol drinking and smoking Were different**   1. Case P, Ng Fat L, Shelton N. Exploring the characteristics of newly defined at-risk drinkers following the change to the UK low risk drinking guidelines: a retrospective analysis using Health Survey for England data. BMC Public Health. 2019 Dec;19(1):1-3. 2. Iversen A, Waterdrinker A, Fear N, Greenberg N, Barker C, Hotopf M, Hull L, Wessely S. Factors associated with heavy alcohol consumption in the UK armed forces: data from a health survey of Gulf, Bosnia, and era veterans. Military medicine. 2007 Sep 1;172(9):956-61. 3. Fear NT, Iversen A, Meltzer H, Workman L, Hull L, Greenberg N, Barker C, Browne T, Earnshaw M, Horn O, Jones M. Patterns of drinking in the UK Armed Forces. Addiction. 2007 Nov;102(11):1749-59. 4. Cunha NO, Giatti L, Assunção AÁ. Factors associated with alcohol abuse and dependence among public transport workers in the metropolitan region of Belo Horizonte. International archives of occupational and environmental health. 2016 Aug;89(6):881-90. 5. Tevik K, Selbæk G, Engedal K, Seim A, Krokstad S, Helvik AS. Factors associated with alcohol consumption and prescribed drugs with addiction potential among older women and men–the Nord-Trøndelag health study (HUNT2 and HUNT3), Norway, a population-based longitudinal study. BMC geriatrics. 2019 Dec;19(1):1-5. 6. Machado ÍE, Lana FC, Felisbino-Mendes MS, Malta DC. Factors associated with alcohol intake and alcohol abuse among women in Belo Horizonte, Minas Gerais State, Brazil. Cadernos de saúde pública. 2013; 29:1449-59. 7. Kim SS, Lee HO, Kiang P, Kalman D, Ziedonis DM. Factors associated with alcohol problems among Asian American college students: gender, ethnicity, smoking and depressed mood. Journal of Substance Use. 2014 Mar 1;19(1-2):12-7. 8. Liu YC, Chen HH, Lee JF, Chu KH, Chien LY. Factors associated with drinking behavior among immigrant women in Taiwan. Substance Use & Misuse. 2017 Apr 16;52(5):674-82. 9. Ojo OA, Louwagie G, Morojele N, Rendall-Mkosi K, London L, Olorunju S, Davids A. Factors associated with female high-risk drinking in a rural and urban South African site. South African medical journal. 2010 Mar 1;100(3):180-2. 10. Hongthong D, Somrongthong R, Wongchaiya P, Kumar R. Factors predictive of alcohol consumption among elderly people in a rural community: a case study in Phayao Province Thailand. Journal of Ayub Medical College Abbottabad. 2016 May 31;28(2):237-40. 11. Thomas JL, Renner CC, Patten CA, Decker PA, Utermohle CJ, Ebbert JO. Prevalence and correlates of tobacco use among middle and high school students in western Alaska. International journal of circumpolar health. 2010 Apr 26;69(2):168-80. 12. Costa JS, Silveira MF, Gazalle FK, Oliveira SS, Hallal PC, Menezes AM, Gigante DP, Olinto MT, Macedo S. Heavy alcohol consumption and associated factors: a population-based study. Revista de saúde pública. 2004; 38:284-91. 13. Crum RM, Chan YF, Chen LS, Storr CL, Anthony JC. Incidence rates for alcohol dependence among adults: prospective data from the Baltimore Epidemiologic Catchment Area Follow-Up Survey, 1981-1996. Journal of studies on alcohol. 2005 Nov;66(6):795-805. 14. Medina E, Kaempffer AM, Cornejo E, Hernández E. Smoking in Santiago, 1993-94. Revista Medica de Chile. 1995 May 1;123(5):652-8. 15. Grant BF. Age at smoking onset and its association with alcohol consumption and DSM-IV alcohol abuse and dependence: results from the National Longitudinal Alcohol Epidemiologic Survey. Journal of substance abuse. 1998 Jan 1;10(1):59-73. 16. Jensen MK, Sørensen TI, Andersen AT, Thorsen T, Tolstrup JS, Godtfredsen NS, Grønbaek MN. A prospective study of the association between smoking and later alcohol drinking in the Danish population. Ugeskrift for Laeger. 2004 Oct 1;166(42):3718-22. 17. Tobias JS, da Silva DL, Ferreira PA, da Silva AA, Ribeiro RS, Ferreira AS. Alcohol use and associated factors among physicians and nurses in northeast Brazil. Alcohol. 2019 Mar 1; 75:105-12. 18. Peltzer K, Pengpid S. Alcohol use and problem drinking in South Africa: Results from a national-population-based survey 2014-2015. Journal of Psychology in Africa. 2018 Mar 4;28(2):147-51. 19. Ekuklu G, Deveci S, Eskiocak M, Berberoglu U, Saltik A. Alcoholism prevalence and some related factors in Edirne, Turkey. Yonsei Medical Journal. 2004 Apr 1;45(2):207-14. 20. Lin YP, Hsieh HI, Chen YC, Cheng TJ. Association between smoking, acetaldehyde dehydrogenase-2 1-1 status, and alcohol drinking among Taiwanese polyvinyl chloride workers. Journal of occupational and environmental medicine. 2001 Aug 1:701-5. 21. Lee EK, Kim OS, Hong JY. Characteristics and factors associated with problem drinking in male workers. Asian nursing research. 2015 Jun 1;9(2):132-7. 22. Schutte KK, Brennan PL, Moos RH. Predicting the development of late‐life late‐onset drinking problems: a 7‐year prospective study. Alcoholism: Clinical and Experimental Research. 1998 Sep;22(6):1349-58. 23. Gottlieb Hansen AB, Hvidtfeldt UA, Grønbæk M, Becker U, Søgaard Nielsen A, Schurmann Tolstrup J. The number of persons with alcohol problems in the Danish population. Scandinavian journal of public health. 2011 Mar;39(2):128-36. 24. Stevenson JS, Masters JA. Predictors of alcohol misuse and abuse in older women. Journal of Nursing Scholarship. 2005 Dec;37(4):329-35. 25. Eashwar VA, Gopalakrishnan S, Umadevi R, Geetha A. Pattern of alcohol consumption and its associated morbidity among alcohol consumers in an urban area of Tamil Nadu. Journal of family medicine and primary care. 2019 Jun;8(6):2029. 26. Banta JE, Addison A, Job JS, Yel D, Kheam T, Singh PN. Patterns of alcohol and tobacco use in Cambodia. Asia Pacific Journal of Public Health. 2013 Sep;25(5_suppl):33S-44S. 27. Jonas HA, Dobson AJ, Brown WJ. Patterns of alcohol consumption in young Australian women: socio‐demographic factors, health‐related behaviours and physical health. Australian and New Zealand Journal of Public Health. 2000 Apr;24(2):185-91. 28. Degenhardt L, Hall W. Patterns of co-morbidity between alcohol use and other substance use in the Australian population. Drug and Alcohol Review. 2003 Jan 1;22(1):7-13. 29. Härkönen J, Aalto M, Suvisaari J, Lintonen T, Mäki-Opas T, Peña S, Mäkelä P. Predictors of persistence of risky drinking in adults: an 11-year follow-up study. European addiction research. 2017;23(5):231-7. 30. Nogueira EL, Cataldo Neto A, Cauduro MH, Ulrich LE, Spanemberg L, DeCarli GA, Gomes I. Prevalence and patterns of alcohol misuse in a community-dwelling elderly sample in Brazil. Journal of aging and health. 2013 Dec;25(8):1340-57. 31. John U, Meyer C, Rumpf HJ, Hapke U. Probabilities of alcohol high‐risk drinking, abuse or dependence estimated on grounds of tobacco smoking and nicotine dependence. Addiction. 2003 Jun;98(6):805-14. 32. Young SY, Hansen CJ, Gibson RL, Ryan MA. Risky alcohol use, age at onset of drinking, and adverse childhood experiences in young men entering the US Marine Corps. Archives of pediatrics & adolescent medicine. 2006 Dec 1;160(12):1207-14. 33. McClure LA, Fernandez CA, Clarke TC, LeBlanc WG, Arheart KL, Fleming LE, Lee DJ. Risky drinking in the older population: a comparison of Florida to the rest of the US. Addictive behaviors. 2013 Apr 1;38(4):1894-7. 34. Taylor M, Knox J, Chhagan MK, Kauchali S, Kvalsvig J, Mellins CA, Arpadi SM, Craib MH, Davidson LL. Screening caregivers of children for risky drinking in KwaZulu-Natal, South Africa. Maternal and child health journal. 2016 Nov;20(11):2392-401. 35. Park SH, Kim CH, Kim DJ, Suk KT, Park HY, Lee JG, Shin KJ, Park JH, Kim TO, Yang SY, Moon YS. Secular trends in prevalence of alcohol use disorder and its correlates in Korean adults: results from Korea National Health and Nutrition Examination Survey 2005 and 2009. Substance abuse. 2012 Oct 1;33(4):327-35. 36. Liu R, Chen L, Zhang F, Zhu R, Lin X, Meng X, Li H, Lei X, Zhao Y. Trends in alcohol intake and the association between socio-demographic factors and volume of alcohol intake amongst adult male drinkers in China. International journal of environmental research and public health. 2019 Feb;16(4):573. 37. Norberg M, Malmberg G, Ng N, Broström G. Use of moist smokeless tobacco (snus) and the risk of development of alcohol dependence: a cohort study in a middle-aged population in Sweden. Drug and alcohol dependence. 2015 Apr 1; 149:151-7. 38. Fávero JL, Meucci RD, Faria NM, Fiori NS, Fassa AG. Alcohol consumption among tobacco farmers: prevalence and associated factors. Ciência & Saúde Coletiva. 2018; 23:871-82. 39. Junqueira MA, Ferreira MC, Soares GT, Brito IE, Pires PL, Santos MA, Pillon SC. Alcohol use and health behavior among nursing professionals. Revista da Escola de Enfermagem da USP. 2017 Nov 27;51. 40. Powers JR, Anderson AE, Byles JE, Mishra G, Loxton DJ. Do women grow out of risky drinking? A prospective study of three cohorts of A ustralian women. Drug and alcohol review. 2015 May;34(3):278-88. 41. Yeung SA, Jiang CQ, Zhang WS, Lam TH, Cheng KK, Leung GM, Schooling CM. Systematic differences among never, occasional and moderate alcohol users in southern China, and its use in alcohol research: a cross-sectional study. J Epidemiol Community Health. 2013 Dec 1;67(12):1054-60. 42. Padrão P, Silva-Matos C, Damasceno A, Lunet N. Association between tobacco consumption and alcohol, vegetable and fruit intake across urban and rural areas in Mozambique. Journal of Epidemiology & Community Health. 2011 May 1;65(5):445-53. 43. Marques‐Vidal P, Dias CM. Trends and determinants of alcohol consumption in Portugal: results from the national health surveys 1995 to 1996 and 1998 to 1999. Alcoholism: Clinical and Experimental Research. 2005 Jan;29(1):89-97. 44. McKee SA, Falba T, O’Malley SS, Sindelar J, O’Connor PG. Smoking status as a clinical indicator for alcohol misuse in US adults. Archives of internal medicine. 2007 Apr 9;167(7):716-21. 45. Harrison EL, Desai RA, McKee SA. Nondaily smoking and alcohol use, hazardous drinking, and alcohol diagnoses among young adults: findings from the NESARC. Alcoholism: Clinical and Experimental Research. 2008 Dec;32(12):2081-7. 46. pickering rp, dawson da, grant bf. differences by alcoholic beverage preference: results of the national epidemiologic survey on alcohol and related conditions (nesarc). inalcoholism-clinical and experimental research 2010 jun 1 (vol. 34, no. 6, pp. 180a-180a). commerce place, 350 main st, malden 02148, ma usa: wiley-blackwell publishing, inc. 47. Evren C, Ogel K, Demirci AC, Evren B, Yavuz BG, Bozkurt M. Prevalence of lifetime tobacco, alcohol and drug use among 10th grade students in Istanbul. Klinik Psikofarmakoloji Bülteni-Bulletin of Clinical Psychopharmacology. 2014 Sep 1;24(3):201-10. 48. Manwell LB, Ignaczak M, Czabala JC. Prevalence of tobacco and alcohol use disorders in Polish primary care settings. The European Journal of Public Health. 2002 Jun 1;12(2):139-44. 49. Janghorbani M, Ho SY, Lam TH, Janus ED. Prevalence and correlates of alcohol use: a population‐based study in Hong Kong. Addiction. 2003 Feb;98(2):215-24. 50. Mendoza‐Sassi RA, Béria JU. Prevalence of alcohol use disorders and associated factors: a population‐based study using AUDIT in Southern Brazil. Addiction. 2003 Jun;98(6):799-804. 51. Husky MM, Paliwal P, Mazure CM, McKee SA. Gender differences in association with substance use diagnoses and smoking. Journal of addiction medicine. 2007 Sep 1;1(3):161-4. 52. Davoren MP, Shiely F, Byrne M, Perry IJ. Hazardous alcohol consumption among university students in Ireland: a cross-sectional study. BMJ open. 2015 Jan 1;5(1):e006045. 53. Merrick EL, Horgan CM, Hodgkin D, Garnick DW, Houghton SF, Panas L, Saitz R, Blow FC. Unhealthy drinking patterns in older adults: prevalence and associated characteristics. Journal of the American Geriatrics Society. 2008 Feb;56(2):214-23. 54. Pomerleau J, McKee M, Rose R, Haerpfer CW, Rotman D, Tumanov S. Hazardous alcohol drinking in the former Soviet Union: a cross-sectional study of eight countries. Alcohol & alcoholism. 2008 May 1;43(3):351-9. 55. Wang MH, Lee CH, Lai CY, Chueh KH, Yen CF, Yang MS. Harmful alcohol use among aboriginal people in southern Taiwan: The prevalence, correlates, and adverse drinking effects. Journal of addictions nursing. 2014 Jan 1;25(1):41-7. 56. Vellios NG, Van Walbeek CP. Self-reported alcohol use and binge drinking in South Africa: Evidence from the National Income Dynamics Study, 2014-2015. South African Medical Journal. 2018 Feb 8;108(1):33-9. |
| **Not Reported Appropriate Effect Size**   1. Banta JE, Przekop P, Haviland MG, Pereau M. Binge drinking among California adults: results from the 2005 California Health Interview Survey. The American journal of drug and alcohol abuse. 2008 Jan 1;34(6):801-9. 2. Pengpid S, Vonglokham M, Kounnavong S, Sychareun V, Peltzer K. Concurrent binge drinking and current tobacco use and its social and health correlates among adults in Laos. Journal of Human Behavior in the Social Environment. 2019 Apr 3;29(3):403-14. 3. Ilomäki J, Gnjidic D, Le Couteur DG, Bell JS, Blyth FM, Handelsman DJ, et al. Alcohol consumption and tobacco smoking among community‐dwelling older A ustralian men: The Concord Health and Ageing in Men Project. Australas. J. Ageing. 2014;33(3):185-92. |
| **Different Study Type (Two Cohorts Study and One Cas-Control Study)**   1. Longitudinal Trajectories of Heavy Drinking in Adults in The United States of America. 2. Tan JH, Shahwan S, Satghare P, Cetty L, Verma S, Sendren JR, Chong SA, Subramaniam M. Binge drinking: Prevalence, correlates, and expectancies of alcohol use among individuals with first‐episode psychosis. Early intervention in psychiatry. 2019 Oct;13(5):1136-45. 3. Kim S, Rifkin S, John SM, Jacob KS. Nature, prevalence and risk factors of alcohol use in an urban slum of Southern India. The National medical journal of India. 2013 Jul;26(4):203. |
