# Supplementary material for: The association between current smoking and binge drinking among adults: A systematic review and meta-analysis of cross-sectional studies
Source: Front Psychiatry. 2023 Jan 18;13:1084762. doi: 10.3389/fpsyt.2022.1084762 (PMC9889925; doi:10.3389/fpsyt.2022.1084762)
Supplement: Supplementary file 4 [file Table_4.docx]

**Table S4: Sensitivity analysis with metaninf command on the association between binge drinking and current Smoking in both sexes.**

| Study Omitted | Adjusted Odds Ratio  (AOR) | Lower | Upper |
| --- | --- | --- | --- |
| Jean H. Kim (2008) | 2.87 | 1.90 | 4.35 |
| Monideepa B. Becerra (2013)  Vietnamese-American | 2.74 | 1.81 | 4.16 |
| Monideepa B. Becerra (2013)  South Asian-American | 3.09 | 2.02 | 4.73 |
| Monideepa B. Becerra (2013)  Japanese-American | 2.88 | 1.87 | 4.44 |
| Monideepa B. Becerra (2013)  Chinese-American | 2.99 | 1.92 | 4.68 |
| Monideepa B. Becerra (2013)  Korean-American | 3.00 | 1.95 | 4.62 |
| Monideepa B. Becerra (2013)  Filipino-American | 2.96 | 1.90 | 4.61 |
| Ha-Na Kim (2014) | 2.78 | 1.90 | 4.06 |
| Francesco Bartoli (2014) | 3.28 | 2.30 | 4.70 |
| Roopali Bipin Parikh (2015) | 3.17 | 2.09 | 4.82 |
| Noah R. Gubner (2016) | 3.15 | 2.06 | 4.82 |
| EO Owolabi (2018) | 2.77 | 1.82 | 4.23 |
| Combined | 2.97 | 1.98 | 4.45 |
